# An Ecological Analysis of HPV Vaccination in the United States Before and During the COVID‐19 Pandemic by Age, Sex, and Urbanicity Using Private Insurance Claims Data

**DOI:** 10.1002/cam4.70761

**Published:** 2025-03-21

**Authors:** Milkie Vu, Jingjing Li, Kai Hong, Jennifer W. Kaminski, Bo‐Hyun Cho, Yoonjae Kang

**Affiliations:** ^1^ Department of Preventive Medicine, Feinberg School of Medicine Northwestern University Chicago Illinois USA; ^2^ Department of Behavior, Social, and Health Education Sciences, Rollins School of Public Health Emory University Atlanta Georgia USA; ^3^ Office of Policy, Performance, and Evaluation Centers for Disease Control and Prevention Atlanta Georgia USA; ^4^ The National Center for Immunization and Respiratory Diseases Centers for Disease Control and Prevention Atlanta Georgia USA

## Abstract

**Introduction:**

We aim to assess HPV vaccine administration among privately insured populations before and during the COVID‐19 pandemic in the United States and stratify the assessments by demographic and geographic characteristics.

**Methods:**

Using the Merative MarketScan Commercial Claims and Encounters Database, we estimated monthly and yearly HPV vaccine administration among people aged 9–26 from 2019 to 2022, measured as the proportion of the enrolled population who received ≥ 1 dose of HPV vaccine during that month or year, and their relative percent change from 2020 to 2022, compared to the same period in 2019, overall and stratified by age group, sex, and urbanicity.

**Results:**

HPV vaccine administration in 2020, 2021, and 2022 was lower than in 2019 and continued to decline for all age groups. The relative percent change in rate in 2022 relative to 2019 was −6.0% among children, −38.3% among adolescents, and −42.5% among young adults. The patterns were similar across subgroups, with certain disparities in magnitude. By subpopulations, the highest percent declines in 2022 relative to 2019 in each age group were observed among children in rural areas (−13.5%), male adolescents (−39.8%), and young adults in rural areas (−46.0%).

**Conclusion:**

During the COVID‐19 pandemic, HPV vaccine administration dropped substantially and had not exceeded the pre‐pandemic levels by the end of 2022, with larger declines seen among male adolescents and young adults in rural areas. Our results highlight the need for continuing monitoring and targeted intervention strategies to improve HPV vaccine administration.

## 1 Introduction

Human papillomavirus (HPV) vaccine provides protection against more than 90% of HPV‐attributable cancers [1, 2]. HPV vaccine is recommended for routine vaccination at age 11 or 12 years, with a second dose given 6–12 months after the first dose [3]. It can be given to children as young as 9 years [3]. Despite its benefits, HPV vaccination coverage in the United States lags behind that of other recommended adolescent vaccines and is below the Healthy People 2030 target of 80% [4].

Following the declaration of the COVID‐19 public health emergency (PHE), model‐based analyses predicted that the pandemic would lead to significant declines in HPV vaccine administration in the United States [5, 6] and that substantial catch‐up efforts in the next 2 to 10 years would be required [6]. A study using state immunization data from 10 jurisdictions found a decrease in HPV vaccination for children and adolescents from March to September 2020 compared to the same time periods in 2018 and 2019 [7]. Data from the National Immunization Survey‐Teen show that overall HPV vaccination rates for adolescents aged 13 to 17 in 2022 and 2023 were similar to pre‐pandemic periods, with the exception of certain groups (e.g., those insured by Medicaid or Vaccines for Children‐eligible children) [8, 9]. A study of a predominantly Hispanic community in the border region of Texas observed a decrease in HPV vaccine initiation rates among children and adults during the pandemic (2020–2021), which improved slightly in the post‐pandemic period (2022–2023), though they did not return to the pre‐pandemic (2016–2019) levels [10]. Studies using electronic medical charts from health systems have shown mixed evidence regarding the impact of the pandemic. One study reports a decrease in adolescent HPV vaccination in the pandemic period compared to the pre‐pandemic period [11]. Meanwhile, others found that administered HPV vaccine doses have returned to pre‐pandemic levels [12] or that the percentage of patients receiving HPV vaccination was higher in 2021 compared to 2019 [13].

Different restrictions and non‐pharmacological measures of pandemic control also likely impacted HPV vaccination. Structural changes in healthcare delivery, such as the shift to telehealth visits, as well as patient concerns about exposure to COVID‐19, may have reduced the number of visits to healthcare facilities and limited opportunities for vaccination [14]. Many U.S. primary care clinics also operated at reduced capacity during the pandemic to accommodate social distancing measures and implement stricter cleaning protocols, which may have affected their ability to provide routine vaccination [15, 16]. Moreover, although no studies have directly examined how the introduction of COVID‐19 vaccination influenced clinics' prioritization of HPV vaccination, research has documented an increase in HPV vaccine hesitancy during the COVID‐19 pandemic [17].

Little is known on whether the effects of the pandemic on HPV vaccination differed by sociodemographic characteristic, particularly in privately insured populations. This focus is important, as more than half of children under the age of 18 in the United States are covered by private insurance [18] and some research has shown that adolescents with private insurance also have lower HPV vaccine uptake compared to those covered by Medicaid [19, 20]. A better understanding of how the COVID‐19 pandemic affected HPV vaccination may inform targeted outreach and messaging efforts for this substantial population. In this study, we used nationwide medical claims data for privately insured populations to investigate HPV vaccine administration before and during the PHE. We examined whether these rates differed by age group, sex, and urbanicity.

## 2 Methods

### 2.1 Study Design and Variables

This ecological study (aggregated from data at the individual level) is reported following the STROBE reporting guideline [21]. We used data from January 2019 to December 2022 in the Merative MarketScan Commercial Claims and Encounters Database, a large nationwide convenience sample of medical claims data among approximately 80 million enrollees who had commercial employer‐sponsored health insurance in the United States. The data contained enrollees' demographics, including age, sex, urbanicity (i.e., rural vs. non‐rural residence, defined by MarketScan as not living in a metropolitan statistical area vs. living in a metropolitan statistical area), health insurance plan enrollment details, and medical claims data such as diagnoses, procedures, and prescriptions [22]. For each calendar year, we identified enrollees who received the HPV vaccine based on the presence of CPT codes specific for HPV vaccines: 90649, 90,650, and 90,651. Monthly claims for vaccination were identified by procedure codes on claims with a service date within the month examined. The denominator populations were the people aged 9–26 who were continuously enrolled in the health insurance plans in the corresponding calendar year (i.e., 365 days in 2019, 2021, or 2022, or 366 days in 2020).

### 2.2 Statistical Procedures

Monthly and yearly vaccine administration were calculated as the proportion of the denominator population who received ≥ 1 dose of HPV vaccine during that month or year. SAS (version 9.4; SAS Institute) was used to describe the samples. We reported HPV vaccine administration in both absolute and relative measures, following the literature on other vaccination during the COVID‐19 pandemic [23]. For absolute measures, we reported HPV vaccine administration (proportion), both monthly and yearly, from 2019 to 2022 by age group (children [9–12 years], adolescents [13–17 years], and young adults [18–26 years]), sex (male, female), and urbanicity (rural and non‐rural residence). Relative measures were used to describe percent changes in HPV vaccines administered during the COVID‐19 pandemic compared to those administered before the pandemic. Data from 2019 were used as baseline to reflect the most recent HPV vaccination before the pandemic and then compared with data from 2020, 2021, and 2022, by age group, sex, and urbanicity. To tease out potential seasonality in HPV vaccination [24], the relative percent change in HPV administration during a month or a year was calculated as the ratio of the administration (proportion) in that month or year to the administration (proportion) during the same corresponding period in 2019, minus one. The trends were tested using univariate linear probability models, with HPV vaccine administration as the dependent variable and the indicator of baseline sample as the independent variable and accounting for potential dependence of samples from different years by clustering the standard errors at the individual level. The confidence intervals for the percent change were obtained by linear interval approximation.

## 3 Results

**FIGURE 1:**
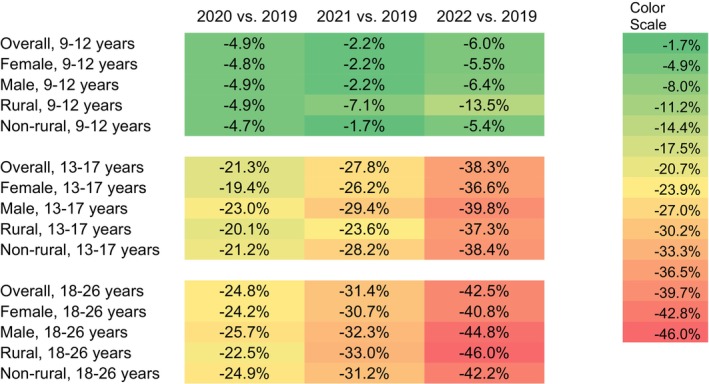
Percent Change in HPV Yearly Vaccination (Proportion) Relative to 2019 by Age, Sex, and Urbanicity, MarketScan CCAE Data, United States, 2019–2022.

Our sample denominator varied over the years, consisting of *n* = 4,819,881 in 2019; *n* = 4,589,165 in 2020; *n* = 4,212,520 in 2021; and *n* = 3,987,173 in 2022 (Table 1), representing about 5.8% of the total U.S. population aged 9 to 26 per the 2020 U.S. Census data (5.7% for 9–12, 5.6 for 13–17, and 6.0% for 18–26) [25]. In 2019, the yearly HPV vaccine administration (proportion) was 24.0%, 12.8%, and 2.8% for children, adolescents, and young adults, respectively, with variations by sex and urbanicity (Table 1). Monthly HPV vaccine administration (proportion) dropped sharply during the months following March 2020 when the PHE was declared, across all age groups, compared with those in the corresponding months in 2019. Overall, we found that the HPV vaccine administration during the PHE in 2020, 2021, and 2022 was lower than prior to the PHE in 2019 and continued to decline (Figure 1). The relative percent change in the overall yearly rate in 2022 relative to 2019 was −6.0% (95% CI [−6.5, −5.4]) among children, −38.3% (95% CI [−38.9, −37.7]) among adolescents, and −42.5% (95% CI [−43.4, −41.5]) among young adults. Among the demographic subgroups defined by sex or urbanicity, the highest percent decreases in each age group were −13.5% (95% CI [−15.3, −11.6]) among children living in rural areas, −39.8% (95% CI [−40.6, −39.0]) among male adolescents, and −46.0% (95% CI [−49.3, −42.8]) among young adults living in rural areas.

**TABLE 1:** Enrollment sample size, yearly HPV vaccine administration (Proportion) among privately insured people aged 9–26 Years, MarketScan CCAE Data, United States, 2019–2022.

	Enrollment sample size				Vaccine administration (%)			
	2019	2020	2021	2022	2019	2020	2021	2022
9–12 years								
Overall	1,025,878	967,340	879,681	819,518	24.0	22.8	23.5	22.6
Female	502,534	473,333	430,301	401,279	24.5	23.4	24.0	23.2
Male	523,344	494,007	449,380	418,239	23.5	22.3	22.9	22.0
Rural	107,544	107,180	93,057	82,296	19.2	18.2	17.8	16.6
Non‐rural	918,334	860,160	786,624	737,222	24.5	23.4	24.1	23.2
13–17 years								
Overall	1,269,087	1,214,348	1,128,609	1,059,740	12.8	10.1	9.3	7.9
Female	622,940	595,437	553,522	520,147	12.6	10.1	9.3	8.0
Male	646,147	618,911	575,087	539,593	13.1	10.1	9.2	7.9
Rural	138,198	139,471	124,411	111,441	9.5	7.6	7.3	6.0
Non‐rural	1,130,889	1,074,607	1,004,198	948,299	13.2	10.4	9.5	8.1
18–26 years								
Overall	2,524,916	2,407,477	2,204,230	2,107,915	2.8	2.1	1.9	1.6
Female	1,255,030	1,196,395	1,092,442	1,045,067	3.4	2.6	2.4	2.0
Male	1,269,886	1,211,082	1,111,788	1,062,848	2.2	1.7	1.5	1.2
Rural	280,333	281,129	248,089	227,845	2.1	1.6	1.4	1.1
Non‐rural	2,244,583	2,126,348	1,956,141	1,880,070	2.9	2.2	2.0	1.7

The most prominent percent declines were in April 2020 when many healthcare practices suggested delaying non‐urgent visits (such as for vaccination) to reduce COVID‐19 transmission: −72.0% (95% CI [−73.7, −70.3]) among children (Appendix A), −76.8% (95% CI: [−78.8, −74.9]) among adolescents (Appendix B), and −71.8% (95% CI [−74.4, −69.2]) among young adults (Appendix C). Thereafter, monthly administration started to recover gradually among all age groups. In the summer of 2021, when the Delta variant of COVID‐19 was dominant in the Unites States, the trend of recovery was disrupted for adolescents and young adults. In 2022, when the Omicron variant of COVID‐19 was widespread, vaccination dropped again across all age groups compared to 2019, with 6.0% (95% CI [5.4, 6.5]) lower administration among children, 38.3% (95% CI: [37.7, 38.9]) lower for adolescents, and 42.5% (95% CI [41.5, 43.4]) lower for young adults (Figure 1).

Greater initial declines and slower recovery in HPV vaccine administration, relative to the baselines in 2019, were observed among male compared to female young adults (Figure 1). For example, HPV vaccine administration in 2021 and 2022 among female young adults was 30.7% (95% CI [29.5, 31.9]) and 40.8% (95% CI [39.6, 42.1]) lower than administration in 2019, while the administration among male young adults in 2021 and 2022 was 32.3% (95% CI [30.7, 33.8]) and 44.8% (95% CI [43.4, 46.3]) lower, respectively (Figure 1). A different pattern was observed for children: compared to 2019, administration among female and male children was 2.2% (95% CI [1.4, 2.9]) and 2.2% (95% CI [1.5, 3.0]) lower in 2021, respectively, and 5.5% (95% CI [4.8, 6.2]) and 6.4% (95% CI [5.7, 7.1]) lower in 2022, respectively (Figure 1).

The impact of urbanicity varied by age group. In April 2020, HPV vaccine administration between rural and non‐rural residents decreased with a modest gap among children (69.0% [95% CI [62.8, 75.1]] vs. 72.2% [95% CI [70.5, 73.9]]), with larger gaps between rural and non‐rural residents among adolescents (71.1% [63.9, 78.3] vs. 77.3% [75.3, 79.3]) and young adults (67.4% [58.3, 76.5] vs. 72.2% [69.4, 74.9]) compared to April 2019 (Appendix D). For children and young adults, those in rural areas experienced slower recovery thereafter compared to non‐rural residents. 2022 administration was 13.5% (95% CI [11.6, 15.3]) and 5.4% (95% CI [4.9, 5.9]) lower among rural and non‐rural children compared to 2019, and 46.0% (95% CI [42.8, 49.3]) and 42.2% (95% CI [41.2, 43.2]) lower among rural and non‐rural young adults. The trend is different among adolescents, with 37.3% (95% CI [35.1, 39.5]) lower administration in 2022 compared to 2019 among those in rural areas and 38.4% (95% CI [37.8, 39.1]) lower among those in non‐rural areas (Figure 1).

## 4 Discussion

Our findings add important insights to the limited literature on HPV vaccine administration trends during the pandemic. To our knowledge, this study was the first to analyze data from a nationwide sample of privately insured populations and provide estimates by sex, age groups, and urbanicity. The monthly administration of HPV vaccine doses dropped after the declaration of the PHE across all age groups. While administration was comparable to or even higher during some months of the PHE among children following an initial decline, these trends were not observed among adolescents and young adults. In 2022, HPV vaccine administration did not exceed the 2019 level for any age group. In addition, older males, rural children, and rural young adults, despite small samples from rural areas, consistently experienced large declines (or slow recoveries) in administration from 2021 to 2022 compared to their counterparts in the same age group.

Our findings align with studies documenting the declines in HPV vaccination following the PHE declaration. For example, one found a decrease in HPV vaccination for children and adolescents from March to September 2020 compared to the same time periods in 2018 and 2019 [7]. Meanwhile, another study using data from children aged 9 to 14 at pediatric practices found that a smaller percentage of children were vaccinated during the pandemic (March 2020 to April 2022) compared to the pre‐pandemic period (March 2018 to February 2020) and that when COVID‐19 positivity rates peaked, HPV vaccination also declined [11]. However, it should also be noted that these studies did not include young adults. They also provided neither estimates by age group, sex, or urbanicity nor focused exclusively on privately insured populations. Differences in the timeframes used to define pandemic or post‐pandemic periods also complicate comparisons across studies.

Data from the National Immunization Survey–Teen highlight additional trends among adolescents aged 13 to 17 [8]. Between 2015 and 2021, coverage with ≥ 1 HPV vaccine dose was lower among those with private insurance compared to those covered by Medicaid [8]. However, in 2022, because coverage declined among Medicaid beneficiaries while remaining stable among those with private insurance, this change resulted in similar coverage between the two groups during this year [8]. In addition, in contrast to our findings, a study of children aged 9 to 12.9 years in California found that, following an initial decrease in March to April 2020, the number of HPV vaccine doses administered in 2020 and 2021 has returned to levels seen in 2019 [12]. However, these studies relied on either nationally representative survey data or electronic medical records from a single health system and focused on narrower age ranges compared to our study, which used medical claims data and included individuals aged 9 to 26. These methodological and population differences provide important context for understanding the variation in findings across studies.

Declines in HPV vaccine administration, as documented in our study, could hamper long‐term efforts that have been made toward improving HPV vaccination coverage and pose critical challenges to population‐level reductions of HPV‐related cancers [15]. Communication efforts and other evidence‐based strategies that focus on adolescents/parents of adolescents and young adults (particularly males for both groups), including standing orders and reminders/recalls, may help reverse the observed decline in HPV vaccination [26, 27]. Moreover, rural areas have historically experienced lower HPV vaccination coverage [8, 28]. The pandemic has compounded challenges for rural clinics in implementing interventions to increase HPV vaccination [14]. HPV vaccine ordering has recovered post‐pandemic but still showed cumulative deficits, with rural counties experiencing greater shortfalls compared to urban areas [29]. Continued monitoring of uptake based on geographic residence could inform policies or interventions to promote HPV vaccination.

### 4.1 Strengths and Limitations

Strengths of the study include the use of a large, nationwide sample of medical claims data and the focus on sex and urbanicity within each age group (children, adolescents, and young adults). However, as MarketScan Commercial databases only include claims submitted to participating employer‐sponsored health insurance plans, our findings cannot be generalized to people with public health insurance or no insurance. Though we use an extensive database of claims data, we might not have captured all HPV vaccination in this population, especially if people received vaccines outside of the healthcare system covered by their private insurance (e.g., school‐based or community events). As we focused on administration and our data did not distinguish the first, second, or third dose, our results could be different from the trends of coverage or series completion.

## 5 Conclusion

Using nationwide medical claims data for privately insured populations, we provide critical insights into HPV vaccination before and during the COVID‐19 pandemic by age, sex, and urbanicity. As of the end of 2022, the yearly vaccine administration did not exceed pre‐pandemic levels, with larger declines seen among adolescents and young adults. Findings underscore the need for targeted communication and intervention strategies, especially in rural areas and among older males, to counteract the declines and ultimately improve HPV vaccination coverage and reduce HPV‐related cancers.

## Author Contributions

M.V. as the corresponding and submitting author, contributed to the writing of the original draft, funding acquisition, project administration, visualization, and reviewing and editing of the manuscript. J.L. contributed to validation and writing review and editing. K.H. contributed to the methodology, visualization, validation, writing review and editing, software development, formal analysis, and data curation. J.W.K. contributed to writing review and editing. B.‐H.C. contributed to writing review and editing. Y.K. contributed to project administration, writing review and editing.

## Conflicts of Interest

The authors declare no conflicts of interest.

## Data Availability

Mirative Marketscan databases are commercial datasets and are only accessible to users and organizations that purchase the access.

## Appendix D Enrollment Sample Size, April HPV Vaccine Administration (Proportion) Among Privately Insured People Aged 9–26 Years, MarketScan CCAE Data, United States, 2019–2022

**Table:**

	Enrollment Sample Size				Vaccine Administration (%)			
	2019	2020	2021	2022	2019	2020	2021	2022
9–12 years								
Overall	1,025,878	967,340	879,681	819,518	1.7	0.5	1.6	1.5
Female	502,534	473,333	430,301	401,279	1.7	0.5	1.7	1.6
Male	523,344	494,007	449,380	418,239	1.6	0.5	1.6	1.4
Rural	107,544	107,180	93,057	82,296	1.2	0.4	1.1	1.0
Non‐rural	918,334	860,160	786,624	737,222	1.7	0.5	1.7	1.6
13–17 years								
Overall	1,269,087	1,214,348	1,128,609	1,059,740	1.0	0.2	0.8	0.6
Female	622,940	595,437	553,522	520,147	1.0	0.2	0.8	0.6
Male	646,147	618,911	575,087	539,593	1.0	0.2	0.7	0.6
Rural	138,198	139,471	124,411	111,441	0.7	0.2	0.5	0.4
Non‐rural	1,130,889	1,074,607	1,004,198	948,299	1.0	0.2	0.8	0.6
18–26 years								
Overall	2,524,916	2,407,477	2,204,230	2,107,915	0.3	0.1	0.2	0.2
Female	1,255,030	1,196,395	1,092,442	1,045,067	0.4	0.1	0.2	0.2
Male	1,269,886	1,211,082	1,111,788	1,062,848	0.2	0.1	0.1	0.1
Rural	280,333	281,129	248,089	227,845	0.2	0.1	0.1	0.1
Non‐rural	2,244,583	2,126,348	1,956,141	1,880,070	0.3	0.1	0.2	0.2
